# Hybrid Carbon Nanocomposites Made of Aerospace-Grade Epoxy Showing Synergistic Effects in Electrical Properties and High Processability

**DOI:** 10.3390/polym15051163

**Published:** 2023-02-25

**Authors:** Federica Zaccardi, Elisa Toto, Fabrizio Marra, Maria Gabriella Santonicola, Susanna Laurenzi

**Affiliations:** 1 Department of Astronautical Electrical and Energy Engineering, Sapienza University of Rome, Via Salaria 851-881, 00138 Rome, Italy; 2 Department of Chemical Engineering Materials Environment, Sapienza University of Rome, Via del Castro Laurenziano 7, 00161 Rome, Italy

**Keywords:** nanocomposites, carbon nanotubes, graphene, electrical properties, rheological properties, processability

## Abstract

In this work, we investigate the processability and the volumetric electrical properties of nanocomposites made of aerospace-grade RTM6, loaded with different carbon nanoparticles. Nanocomposites with graphene nanoplatelets (GNP), single-walled carbon nanotubes (SWCNT) and hybrid GNP/SWCNT in the ratio 2:8 (GNP_2_SWCNT_8_), 5:5 (GNP_5_SWCNT_5_) and 8:2 (GNP_8_SWCNT_2_) were manufactured and analyzed. The hybrid nanofillers are observed to have synergistic properties as epoxy/hybrid mixtures showed better processability than epoxy/SWCNT, while maintaining high values of electrical conductivity. On the other hand, epoxy/SWCNT nanocomposites present the highest electrical conductivities with the formation of a percolating conductive network at lower filler content, but very large viscosity values and filler dispersion issues, which significantly affect the final quality of the samples. Hybrid nanofiller allows us to overcome the manufacturing issues typically associated with the use of SWCNTs. The combination of low viscosity and high electrical conductivity makes the hybrid nanofiller a good candidate for the fabrication of aerospace-grade nanocomposites with multifunctional properties.

## 1. Introduction

In recent years, carbon nanoparticles have attracted considerable interest in several industrial fields for their nature of possessing simultaneously superior physical properties with respect the traditional materials. Indeed, carbon nanotubes may have very high electrical and thermal conductivities, of several orders of magnitude greater than copper [1,2,3], and stronger than steel [4,5,6]. These features make them the ideal candidates for the fabrication of advanced composites with multifunctional properties, including high mechanical, electrical and thermal properties [7,8,9,10]. For example, these materials can be used to realize satellite components by replacing metals with thermally and electrically conductive nanocomposites, thus eliminating the need for active thermal control devices and electrically conductive elements.

The capability of nanocomposites to simultaneously enhance different properties is particularly relevant in the aerospace field, where multifunctional polymer-based composites can be advantageous to satisfy the lightweight requirements of aircrafts and spacecrafts by replacing the heavy and complex subsystems [11,12,13,14,15]. In addition to the structural purposes, the applications of nanocomposite materials may include lightning protection, de-icing, radiation shielding, electrostatic charge dissipation, thermal management and the development of novel sensors [13,16,17]. However, in spite of their exceptional qualities, the use of carbon nanoparticles shows some drawbacks related to their processing. In fact, the enhancement of composite material properties, due to the introduction of carbon nanoparticles, strongly depends on the filler dispersion state [18,19,20], a critical aspect in the fabrication of nanocomposite materials [7,10,21]. Although homogeneous dispersion is an essential requirement for the development of a percolating network, which is responsible for the increase in the electrical and thermal properties of nanocomposites [22] and for the improvement of the fracture toughness [21,23], reaching a proper dispersion state of carbon nanofillers in a polymer blend may be very hard [24]. Indeed, carbon nanotubes, especially single-wall carbon nanotubes, have a strong tendency to form agglomerates and clusters as a consequence of the van der Waals forces [18,20]. Graphene nanoplatelets (GNPs) are generally easier to disperse in a polymer blend with respect to the carbon nanotubes but they may present re-stacking and π–stacking interactions [25]. Surface chemical functionalization of carbon nanoparticles helps to improve their dispersion state in polymer blends [26], yet degradation of the electrical conductivity and mechanical properties might be an issue. Using electrically conductive polymers, such as polyaniline (PANI), to modify nanocarbon surfaces has been shown to increase the electrical conductivity of graphene foams [27,28]. However, the sheet-like shape of the three-dimensional foam may not fit with the stringent through-thickness requirements of aerospace structural components made of long-fiber composite. It has recently been demonstrated that graphene oxide (GO) can be used as surfactant to disperse CNTs due to its high solubility and adhesion of CNTs onto the GO sheets through strong π–π stacking interaction [29,30], exploiting the properties of both carbon allotropic forms. 

From a rheological point of view, nano-reinforced polymer blends may show different rheological behaviors depending on the type of nanofillers used [31,32]. This aspect can strongly limit the use of nanofillers for the realization of structural components and parts. In fact, viscosity plays an important role in industrial processes, where the rheological properties at high shear rates are critical parameters and high viscosity values may compromise the fabrication process of nanocomposites [33]. Ma et al. investigated the rheological response of chemically treated and untreated single-walled carbon nanotubes (SWCNTs) dispersed in an epoxy matrix, reporting high values of steady shear viscosity for the blends containing pristine SWCNTs [18,34]. On the contrary, epoxy resins loaded with multi-walled carbon nanotubes (MWCNTs) or GNPs show lower viscosity values when compared to the SWCNT-loaded ones [35,36,37], and consequently, they show better processability. Thus, even if SWCNTs have higher properties than MWCNTs and GNPs, such as thermal and electrical conductivities [38], the manufacturing processes involving SWCNT-loaded resins are much more limited. Incorporating mixtures of GNPs and CNTs in polymer matrices can create novel hybrid nanocomposites with synergistic capabilities of improving processability and exploiting the properties of both SWCNTs and GNTs. Using CNTs with graphene sheets in epoxy matrices enables the realization of multifunctional nanocomposites with enhanced filler dispersion states, since the restacking of graphene sheets is prevented by the presence of interlayered CNTs [39,40,41]. The carbon nanotubes form connections among the graphene sheets, thus allowing the realization of a conductive network which leads to an enhancement of the electrical and thermal properties. The improvement of the electrical and thermal properties combined with the good degree of dispersion that can be achieved makes the use of hybrid fillers especially suited for the realization of nanocomposite films. Manufacturing techniques such as doctor blade [42], spray coating [43] and spin coating [11] could be used for the fabrication of hybrid films for thermal management (thermal stress mitigation and passive thermal control), electrostatic charge mitigation, and electromagnetic shielding purposes, preventing spacecraft and aircraft failures by simultaneously saving weight. An improvement of the mechanical properties has also been observed; however, the ratio of GNPs and CNTs has a significant impact on the overall nanocomposite properties [36,39,40,44,45]. Chatterje et al. researched the properties of MWCNT/GNP epoxy nanocomposites identifying a significant improvement in the flexural modulus when mixing the nanofillers in the ratios of 9:1 and 5:1 [39]. On the contrary, Yang et al. achieved the greatest improvement in the mechanical and thermal properties when mixing functionalized MWCNTs and GNPs in the opposite ratio of 1:9 [40]. Yue et al. studied the properties of epoxy nanocomposites loaded with different ratios of MWCNTs and GNPs, observing that the combination of MWCNT to GNP in the ratio of 8:2 is able to synergistically enhance the mechanical and electrical properties [41].

The aim of this work is to investigate the potentiality of hybrid nanocarbon systems to overcome the problems of nanocomposite processing, such as poor filler dispersion and increase in viscosity, which typically makes the use of SWCNTs inapplicable in industrial processes despite their exceptional properties. The point of view of processing is generally neglected in the literature and the present work aims to fill this gap. An extensive study of the rheological properties of hybrid CNT/GNP fillers dispersed in a typical aerospace resin before curing is reported. This work aims to determine the optimal ratios between graphene and nanotubes to obtain suitable processability characteristics useful for liquid composite molding while enhancing the electrical properties of the nanocomposites. In particular, the properties of aerospace-grade RTM6, a mono-component epoxy resin, loaded with GNP/SWCNT hybrid nanofiller in the ratio of 2:8, 5:5 and 8:2 (hereafter, respectively, referred to as GNP_2_SWCNT_8_, GNP_5_SWCNT_5_ and GNP_8_SWCNT_2_) were investigated in terms of electrical and rheological properties and related to material processability. This resin was selected because it is already qualified for aerospace applications. In addition, it has a low viscosity (0.05 Pa·s) at the injection temperature (about 80 °C) so that it can be used in liquid composite molding to manufacture components with complex geometries and large dimensions [46]. Several studies can be found in the literature on the use of the RTM6 resin for the fabrication of epoxy/MWCNT and epoxy/SWCNT nanocomposites [47,48,49]; yet, to the best of our knowledge, its use in combination with hybrid carbon fillers has not been investigated.

## 2. Materials and Methods

### 2.1. Materials

Single-walled carbon nanotubes with a purity higher than 85% were purchased from OCSiAl Europe (Leudelange, Luxembourg). According to the producer datasheet, the average diameter is ~1.8 nm and the average length is >5 µm. Graphene nanopowder of grade AO-4 was from Graphene Supermarket (flake thickness 60 nm, particle lateral size ≤ 7 µm, specific surface area (SSA) ≤40 m^2^ g^−1^) and exfoliated graphene nanoplatelets (xGNP) with different specific surface area and aspect ratio were purchased from XG Sciences (East Lansing, MI, USA). In particular, nanoplatelets of grade M5 (average thickness 6–8 nm, average diameter 5 µm, SSA 120–150 m^2^ g^−1^), grade H5 (average thickness 15 nm, average diameter 5 µm, SSA 50–80 m^2^ g^−1^), and grade C750 (average thickness ~2 nm, average diameter < 2 µm, SSA ~750 m^2^ g^−1^) were used. The selected resin was the aerospace-grade mono-component epoxy RTM6 (Hexcel, Duxford, UK) formulated for resin-transfer molding processes. All materials were used as received.

### 2.2. Processing of Nanocomposites

Epoxy/SWCNT, epoxy/GNP_2_SWCNT_8_, epoxy/GNP_5_SWCNT_5_ and epoxy/GNP_8_SWCNT_2_ nanocomposites with filler concentrations in the range of 1–5 wt% were prepared. For the hybrid nanofillers, ratios of GNP to SWCNT of 2:8, 5:5 and 8:2 (by weight) were used. Nanocomposite samples with GNP AO-4, xGNP-C750, xGNP-H5 and xGNP-M5, each dispersed in RTM6 epoxy resin at 1 wt%, were also fabricated. The dispersion procedure was tuned considering the dependence of the viscosity from the temperature in order to allow the nanofillers mobility in the resin without incurring premature gelling. In this procedure, the resin was initially pre-heated to 80 °C with a constant rate of 2 °C/min. When the resin was homogenously heated, the desired amount of nanofillers was blended for about 90 min in an ultrasonic bath at 80 °C. According to the RTM6 datasheet, the process window is sufficiently long lasting at 80 °C and the viscosity remains constant during the nanofillers dispersion procedure. During this last step, the mixture was also degassed in order to eliminate any entrapped air bubbles. The same nanoparticle dispersion protocol was used to prepare both nanocomposite mixtures and cured samples for the rheological and electrical testing. The specimens for electrical characterization were realized by pouring the mixture into a silicon mold and curing in an oven at 180 °C for 2 h. The curing parameters were the same for all types of hybrid nanocomposites since the inclusion of 1D fillers does not lead to significant differences in the curing kinetics with respect to the unfilled epoxy mixture [50]. In particular, the increase in the dielectric constant as a function of the curing kinetics is in order of 10^−2^ [51] whereas the decrease in the dielectric constant due to the presence of carbon nanofiller is several orders of magnitude greater than the effects of the curing kinetics. In addition, Li et al. show that the effect of the curing time is negligible after a certain time, and thus it is not convenient to extend the cure after that.

### 2.3. Characterization Methods

The dispersion state of the nanofillers was investigated using a quantitative method based on the analysis of grayscale optical images [52]. Droplets of the nanocomposite fluids were placed between microscopy slides and imaged in transmitted light using the high-resolution video camera of a DataPhysics OCA15Pro instrument. The calculated dispersion index (I_d_) was averaged over 10 different images across each sample. SEM investigations were carried out using a VEGA II LSH instrument (Tescan, Brno, Czech Republic) at an accelerating voltage of 5 kV and 40× magnification. SEM images were processed using the MountainsMap 7 software (Digital Surf, Besançon, France), which allows a three-dimensional (3-D) reconstruction of the specimen surface and the evaluation of surface roughness from images acquired at different tilt angles (0° and 5°). The roughness was averaged over values measured on profiles extracted every 0.25 mm across the reconstructed 3-D surface. The analysis was performed on the top surface of the specimen, which was exposed to air during the curing process. The electrical conductivity of the cured nanocomposite samples was determined by electrical impedance spectroscopy (EIS) over the frequency range 20 Hz–1 MHz using an Agilent E4980A Precision LCR Meter. The samples (10 mm × 10 mm) were placed in a custom-made Teflon cell with two square copper electrodes measuring 10 mm × 10 mm at the top and bottom surfaces in a two-point configuration. Impedance measurements were performed with the parallel circuit model, providing the equivalent parallel resistance (*R_p_*). The electrical resistance data were averaged over 20 measurements. The volumetric conductivity (*σ_v_*) of the nanocomposites was determined as *σ_v_* = 1/*ρ_v_*, where *ρ_v_* is the volumetric resistivity evaluated according to the ASTM D257-07. The rheological properties of the nanocomposite mixtures were measured using a rotational stress-controlled MCR 302 rheometer (Anton Paar, Austria) equipped with a Peltier heating system. Experiments were conducted in steady shear mode using the 25 mm plate–plate configuration, with gap size in the range of 1.1–1.3 mm depending on the filler concentration. Steady shear measurements were performed at shear rates in the range of 0.1–100 s^−1^ and at 80 °C, the recommended injection temperature for neat RTM6 epoxy in resin transfer molding processes, with a constant heating rate of 2 °C/min starting from room temperature. 

## 3. Results and Discussion

### 3.1. Dispersion and Electrical Properties of RTM6/Carbon Nanocomposites

**Figure 1:**
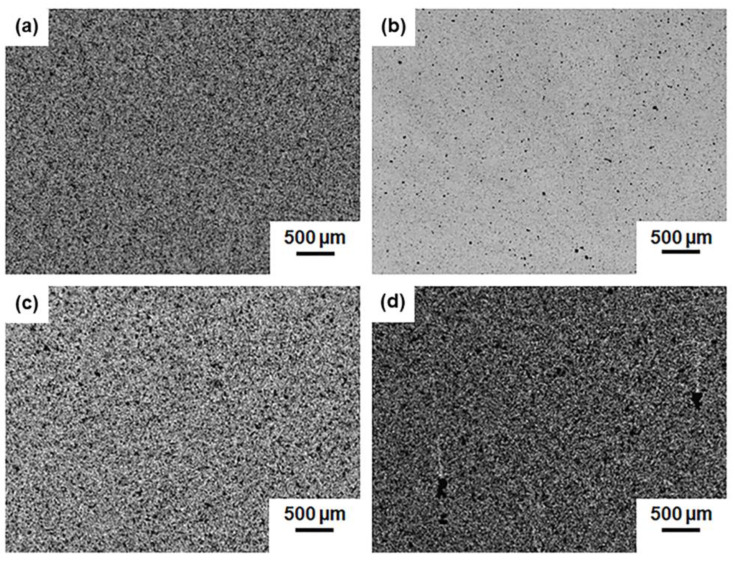
Optical images of (**a**) epoxy/GNP AO-4 (I_d_ = 0.532 ± 0.008), (**b**) epoxy/xGNP-C750 (I_d_ = 0.509 ± 0.015), (**c**) epoxy/xGNP-H5 (I_d_ = 0.478 ± 0.018) and (**d**) epoxy/xGNP-M5 (I_d_ = 0.437 ± 0.013) mixtures after sonication in ultrasonic bath at 80 °C for 90 min.

Optical images showing the dispersion state of the different nanofillers blended into RTM6 resin are in Figure 1. The epoxy/GNP AO-4 system has the best dispersion in comparison with the other nanocomposite fluids, the epoxy/xGNP-C750, epoxy/xGNP-H5 and epoxy/xGNP-M5 systems, which are characterized by similar dispersion degrees. The visual inspection was confirmed by the quantitative analysis of the images leading to dispersion indices (I_d_) with values of 0.532 ± 0.008 for the epoxy/GNP AO-4, 0.509 ± 0.015 for the epoxy/xGNP-C750, 0.478 ± 0.018 for the epoxy/xGNP-H5, and 0.437 ± 0.013 for the epoxy/xGNP-M5 nanocomposites.

The dispersion index was evaluated by comparing the grayscale optical image to the corresponding image with uniformly dispersed pixels. The value of I_d_ is 1 in the ideal case of the uniform dispersion state and decreases as the dispersion state deteriorates [52]. If we look at the producer datasheets, the graphene nanoplatelets of type xGNP-M5, xGNP-H5 and GNP AO-4 all show similar chemical composition with very high purity (carbon content C > 99% and C ~98.5% for the xGNPs and for the GNP AO-4, respectively). For such nanoparticles that differ mainly by their geometrical dimensions, results indicate that the value of the dispersion index is greater when the particle volume is larger and the specific surface area lower. Indeed, it is expected that van der Waals and π-stacking interactions are more effective among particles having high aspect ratios and high specific surface areas. On the other hand, the xGNP-C750 graphene type, despite being the smallest among the nanoparticles considered, has a high dispersion index in the epoxy resin, which can be explained by differences in chemical composition with respect to the other graphene particles. In fact, having about 10% of non-carbon atoms, mainly oxygen (~8%) and nitrogen (~2%), it is possible that the weak non-covalent interactions acting among the xGNP-C750 nanoplatelets are less effective, leading to fewer entanglements [52].

**Figure 2:**
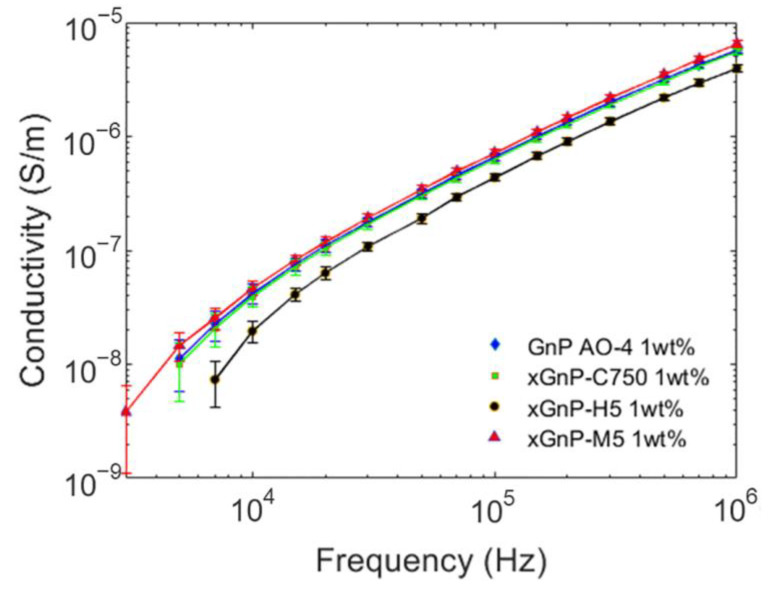
Electrical conductivity of epoxy/GNP nanocomposites containing different grades of GNPs at 1 wt% loading.

The volumetric electrical conductivity of the epoxy/graphene samples at 1 wt% of nanofiller over the frequency range of 1.5 kHz–1 MHz is presented in Figure 2.

All epoxy/graphene samples exhibit the typical behavior of insulating materials, with electrical conductivity that increases with frequency. It is noted that for these nanocomposites with large resistance values at low frequencies, the EIS data were acquired only in the frequency range above 1.5 kHz. This was due to the lower accuracy of the measured resistance values near the instrument measurement limit. For the same reason, having resistivity values higher than 10^10^ Ω·cm, the electrical conductivity of the neat RTM6 epoxy resin is not reported. As shown in Figure 2, there is a small difference in the electrical conductivity values of the investigated graphene-based nanocomposites at all frequencies, except for the epoxy/xGNP-H5 sample which is characterized by lower electrical conductivity than the other nanocomposites. This result can be explained by looking at the geometrical dimensions of the filler as reported in the producer datasheets. In fact, the xGNP-H5 nanofillers have the lowest specific surface area (SSA) and the lowest aspect ratio among the graphene nanoplatelets considered. For this reason, higher concentrations of xGNP-H5 are needed to promote the formation of an efficient interconnected network for the enhancement of electrical properties. As concerns the nanocomposite with GNP AO-4, which has the lowest aspect ratio and SSA among the investigated nanoparticles, the reason for its high electrical conductivity lies in the quality of the dispersion state. 

From the analysis of the electrical and dispersion properties, it emerges that the GNP AO-4 has the best dispersion degree among the nanofillers considered in this study, as well as high electrical conductivity. 

For these reasons, the GNP-AO4 type was selected for the graphene/SWCNT hybrid nanofiller. In particular, the epoxy/hybrid carbon mixtures were prepared with GNP AO-4 and SWCNT in the ratios of 2:8, 5:5 and 8:2 (by weight) (by weight), respectively, referred to as GNP_2_SWCNT_8_, GNP_5_SWCNT_5_ and GNP_8_SWCNT_2_, in order to simultaneously take advantage of the good dispersion state and processability of the GNP AO-4 nanofillers and of the high aspect ratio of SWCNTs. These factors play a crucial role in the development of the 3D conductive network, whilst avoiding the formation of SWCNT entanglements occurring at high loadings of SWCNTs. 

**Figure 3:**
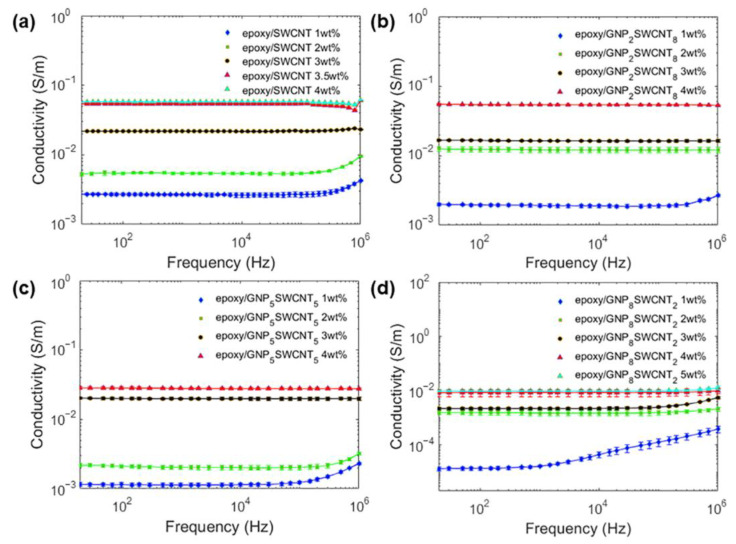
Electrical conductivity of (**a**) epoxy/SWCNT, (**b**) epoxy/GNP_2_SWCNT_8_, (**c**) epoxy/GNP_5_SWCNT_5_ and (**d**) epoxy/GNP_8_SWCNT_2_ nanocomposites with different filler loadings after cure.

Figure 3 shows the volumetric electrical conductivity of the epoxy/SWCNT and hybrid epoxy/GNP_2_SWCNT_8_, epoxy/GNP_5_SWCNT_5_ and epoxy/GNP_8_SWCNT_2_ nanocomposite samples, as determined by electrical impedance spectroscopy over the frequency range 20 Hz–1 MHz. All types of nanocomposites exhibit typical resistive behavior with frequency-independent electrical conductivity up to values of the order of 10^5^ Hz. Overall, the epoxy/SWCNT system has higher electrical conductivities than the epoxy/GNP_8_SWCNT_2_ and epoxy/GNP_5_SWCNT_5_ systems at all concentrations. As regards the epoxy/GNP_2_SWCNT_8_ nanocomposites, they show electrical conductivities close to the values exhibited by the epoxy/SWCNT nanocomposites. In general, the conductivity of nanocomposite systems at high filler concentrations is known to follow the percolation law when a certain volume fraction (percolation threshold) is reached.

**Figure 4:**
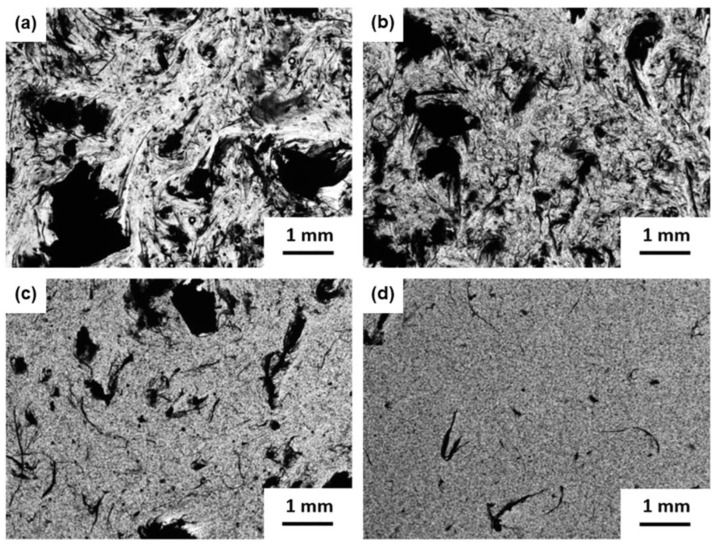
Optical images of (**a**) the epoxy/SWCNT (I_d_ = 0.104 ± 0.007), (**b**) epoxy/GNP_2_SWCNT_8_ (I_d_ = 0.108 ± 0.01), (**c**) epoxy/GNP_5_SWCNT_5_ (I_d_ = 0.201 ± 0.021) and (**d**) epoxy/GNP_8_SWCNT_2_ (I_d_ = 0.443 ± 0.027) mixtures after sonication in ultrasonic bath at 80 °C for 90 min.

The power law from the percolation theory has the following expression [53]:

(1) $$\sigma =\propto {\left(p-p_{c}\right)}^{t}$$

where *p* is the filler volume fraction, *p_c_* is the percolation threshold and *t* is the critical exponent, related to the dimensionality of the system. In theory, the exponent assumes the value of *t* = 1.3 and *t* = 2 for two- and three-dimensional percolating networks, respectively [54,55]. However, different experimental values have been reported [56,57]. For the epoxy/SWCNT systems considered in this work, we are above the percolation threshold, which should be lower than 1 wt% [41,56]. The epoxy/GNP_5_SWCNT_5_ and epoxy/GNP_2_SWCNT_8_ systems also exhibit such behavior, showing an electrical conductivity at 1 wt% almost identical to the epoxy/SWCNT 1wt% system. For the nanocomposites filled with the GNP_8_SWCNT_2_ hybrid, we note the insulating behavior of the 1 wt% mixture at high frequency as well as the remarkable enhancement of electrical properties when increasing the loading fraction up to 2 wt%, meaning that the percolation threshold can be found between these two filler concentrations. In addition, the difference between the electrical conductivity of the epoxy/GNP_8_SWCNT_2_ at 4 wt% and 5 wt% is minimal, meaning that further increasing the nanofiller concentration would be ineffective. By analyzing the results obtained for the epoxy/SWCNT samples at different loadings of filler, we observe a saturation of the electrical conductivity at 3.5 wt% of SWCNTs. The increase in nanotube concentration is not followed by the improvement of the electrical properties, contrary to what would be expected. This behavior can be explained by the increase in entanglements at high filler loadings, which do not participate in the improvement of the network structure and inhibit further improvement of the electrical properties. In addition, the agglomeration phenomenon is responsible for the deterioration of the mechanical properties and for limiting the processability of such nanocomposites. This result suggests that the further increase in the SWCNTs loading fraction over 3.5 wt% is not recommended, and this can be considered an upper limit as concerns the electrical properties of the epoxy/SWCNT nanocomposites. When looking at the volumetric electrical conductivity of the epoxy/GNP_8_SWCNT_2_, epoxy/GNP_5_SWCNT_5_ and epoxy/GNP_2_SWCNT_8_ hybrid samples (Figure 3), it is evident that the epoxy/SWCNT system, except for the epoxy/GNP_2_SWCNT_8_ at 2 wt%, has better electrical properties than the hybrid systems at all mass concentrations due to the presence of the pure SWCNTs with their high aspect ratio. Indeed, the electrical conductivity values of the epoxy/GNP_2_SWCNT_8_, epoxy/GNP_5_SWCNT_5_ and epoxy/GNP_8_SWCNT_2_ hybrid systems are higher than those found by Yue et al. [41], especially at the lowest filler concentrations. This can be explained by considering the high aspect ratio of the selected SWCNTs, which promotes the formation of conductive paths at low filler loadings, but simultaneously favors the aggregation of nanoparticles at high concentrations. As for the GNP-loaded epoxy samples, optical images were used to study the dispersion state of epoxy/SWCNT, epoxy/GNP_2_SWCNT_8_, epoxy/GNP_5_SWCNT_5_ and epoxy/GNP_8_SWCNT_2_ systems with 1 wt% of nanofiller (Figure 4). The epoxy/SWCNT 1wt% sample shows large and interconnected aggregates (Figure 4a), which also explains the high electrical conductivity of the system at 1 wt% of SWCNT. The nanofiller distribution becomes more homogeneous as the GNP:CNT ratio increases, with the epoxy/GNP_8_SWCNT_2_ 1 wt% (Figure 4d) showing the smallest and less interconnected entanglements. To have a better insight into the synergistic effect of the hybrid nanofillers, the dispersion state of the 1 wt% mixtures was quantified in terms of I_d_ [52], with values of 0.103 ± 0.007 for the epoxy/SWCNT, 0.108 ± 0.01 for the epoxy/GNP_2_SWCNT_8_, 0.201 ± 0.021 for the epoxy/GNP_5_SWCNT_5_, and 0.443 ± 0.027 for the epoxy/GNP_8_SWCNT_2_ nanocomposites. Confirming the visual analysis, the epoxy/SWCNT 1 wt% shows the poorest nanofiller distribution. On the contrary, the epoxy/GNP_8_SWCNT_2_ 1 wt% exhibits the highest I_d_ and a more homogeneous nanofiller distribution due to the presence of GNPs, which prevent SWCNTs aggregation. These results suggest that the 2D graphene nanoplatelets are intercalated among the 1D nanotubes leading to the formation of a 3D network of hybrid fillers, which enhances the electrical conductivity of the nanocomposite and prevents the formation of SWCNT agglomerations. This type of structure has been reported in several studies [41,45]. The GO/CNT π–π stacking interactions, which lead to the formation of the intercalated structure, and the GO compatibility with the polar groups of the epoxy resin act synergistically to enhance the dispersion of the hybrid carbon nanofillers in the RTM6 resin. This mechanism is further investigated with the analysis of the rheological behavior, which is reported below.

### 3.2. Rheological Properties of RTM6/carbon Mixtures

**Figure 5:**
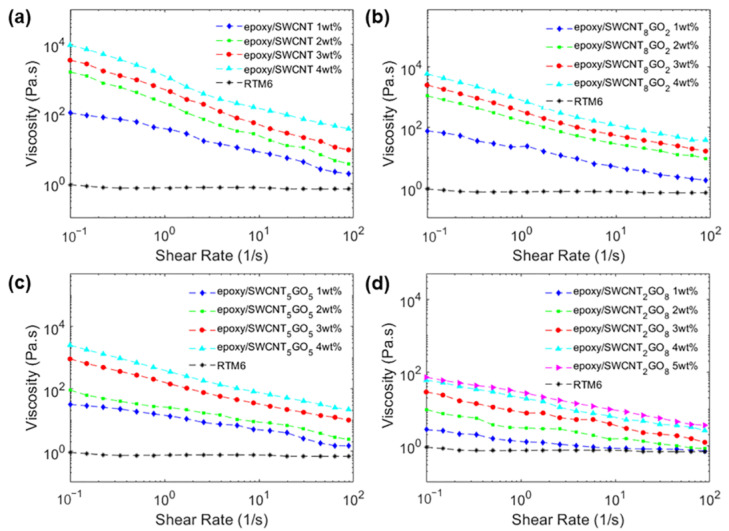
Viscosity of (**a**) epoxy/SWCNT, (**b**) epoxy/GNP_2_SWCNT_8_, (**c**) epoxy/GNP_5_SWCNT_5_ and (**d**) epoxy/GNP_8_SWCNT_2_ mixtures as a function of shear rate measured at the temperature of 80 °C.

The rheological properties of nanocomposite mixtures before cure are known to reflect the internal structure of the suspensions, providing information about the formation of entangled networks, the interactions of the selected fillers with the host matrix, and the processability of the composite fluids. Usually, epoxy resins show a typical Newtonian behavior with the viscosity being independent of the shear rate. On the contrary, it is recognized that GNP- and CNT-loaded epoxy systems exhibit a non-Newtonian shear thinning behavior [18,36,44], with the viscosity that decreases as the shear rate increases due to the shear-induced alignment of the fillers. In fact, during manufacturing processes such as injection or resin-transfer molding, the resin flow is known to orient CNTs and GNPs in the direction of the shear [18,34] and this phenomenon is also expected to occur in hybrid mixtures. Figure 5 shows the steady shear viscosity of the neat RTM6 epoxy resin and of the epoxy/SWCNT, epoxy/GNP_2_SWCNT_8_, epoxy/GNP_5_SWCNT_5_ and epoxy/GNP_8_SWCNT_2_ systems measured at the temperature of 80 °C, thus simulating the injection condition in the resin-transfer molding process.

Except for the neat RTM6 epoxy resin that shows a Newtonian behavior, all mixtures exhibit shear thinning behavior, with the viscosity that decreases with increasing shear rates. For the epoxy/SWCNT samples, the viscosity increases by several orders of magnitude with the increase in nanofiller concentration. The high values of viscosity at low shear rates are attributed to the polymer–particle interactions and to the formation of a structured network of fillers. The particle aspect ratio has a key role in determining the rheological response of the nanocomposite fluids. In fact, longer nanoparticles are expected to interact more easily with each other, forming interconnected structures even at small loading fractions. In our case, SWCNTs with a high aspect ratio (>2500) were selected as nanofillers, which readily increase the viscosity of the composite fluid by interacting with each other. However, as the shear rate increases, the network is de-bundled and the nanotubes align in the shear direction, with the consequence of reducing significantly the viscosity of the system. Whereas the viscosity of the SWCNT-loaded epoxy decreases steeply with increasing shear rates, the shear thinning behavior of the hybrid mixtures becomes less pronounced as the GNP:CNT ratio increases. Indeed, the epoxy/GNP_2_SWCNT_8_ samples have similar viscosity values to those of the SWCNT-loaded epoxy samples. Then, the viscosity decreases in the epoxy/GNP_5_SWCNT_5_ mixtures, with the epoxy/GNP_8_SWCNT_2_ hybrid system having the less pronounced shear thinning behavior.

Different models have been proposed to characterize the rheological behavior of polymer melts. In our case, all the samples analyzed exhibit a shear thinning behavior that can be characterized using the power law model, with the viscosity $\left(\eta \right)$ related to the shear rate ($\dot{\gamma }$) by the following expression [37]:

(2) $$\eta \;\left(\dot{\gamma }\right)=K{\dot{\gamma }}^{n-1}$$

where *K* is the consistency index and *n* is the flow behavior index (dimensionless). The fitted parameters for the RTM6 resin loaded with SWCNTs and with GNP_2_SWCNT_8_, GNP_5_SWCNT_5_ and GNP_8_SWCNT_2_ hybrids are summarized in Table 1.

The consistency index *K* is a proportionality constant and can be considered as the value that the viscosity assumes at a shear rate 1 s^−1^. Depending on the value of the power law index, the fluid exhibits a shear-thickening (*n* > 1), shear-thinning (*n* < 1) or Newtonian behavior (*n* = 1). In addition, the smaller is the value of *n*, the more shear thinning is the fluid. The rheological behaviors of the epoxy/SWCNT and epoxy/GNP_8_SWCNT_2_ mixtures are clearly different, with the second one approaching the Newtonian response more than the first system. More important, the viscosity values of the two nanocomposite mixtures are markedly different: the hybrid system has lower viscosity values than the epoxy/SWCNT system at all concentrations, with variations of one order of magnitude at nanofiller loading of 1 wt% and two orders of magnitude at the higher loadings 4–5 wt% (Figure 5a,d). On the contrary, the rheological behaviors of the epoxy/SWCNT and epoxy/GNP_2_SWCNT_8_ hybrids are similar, showing viscosity values of the same order of magnitude at all nanofiller loadings (Figure 5a,b). As regards the epoxy/GNP_5_SWCNT_5_ system, it simultaneously shows high viscosity values at the high loadings of 3 wt% and 4 wt%, and a rheological behavior that approaches that of the epoxy/GNP_8_SWCNT_2_ system at the nanofiller loadings of 1 wt% and 2 wt% (Figure 5a,c). Overall, by the analysis of the electrical and rheological properties, we can conclude that the epoxy/GNP_8_SWCNT_2_ hybrid system with 3 wt%, 4 wt% and 5 wt% of nanofiller exhibits better electrical properties than the epoxy/SWCNT system at 1 wt% and 2 wt% of SWCNTs, while maintaining lower viscosity values, which translates in better processability for composite manufacturing. Similarly, the epoxy/GNP_5_SWCNT_5_ hybrid system with 1 wt% and 2 wt% of nanofiller exhibits electrical conductivity values of the same order of magnitude as the epoxy/SWCNT system at 1 wt% and 2 wt% of SWCNTs, while maintaining viscosity values one order of magnitude lower. Regarding the epoxy/GNP_2_SWCNT_8_ hybrid system, it shows electrical and rheological behaviors similar to those of the epoxy/SWCNT system, which translates in similar processability for composite manufacturing. 

The rheological behavior of the nanocomposite blends confirms the formation of the 3D filler network in the hybrid systems, where graphene planes are intercalated between 1D single-wall carbon nanotubes. GO platelets can separate the walls of SWCNTs, avoiding the formation of agglomerations among the CNTs. In addition, the graphene layers act as a lubricant reducing the friction between the polymer chains during their relative motion resulting in a decreased polymer viscosity.

**Figure 6:**
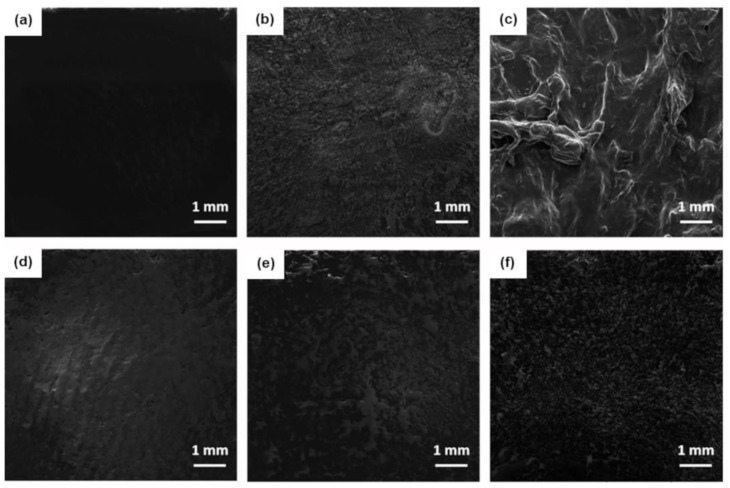
SEM images of (**a**) epoxy/GNP AO-4 1 wt%, (**b**) epoxy/SWCNT 1 wt%, (**c**) epoxy/ SWCNT 4 wt%, (**d**) epoxy/GNP_8_SWCNT_2_ 1 wt%, (**e**) epoxy/GNP_8_SWCNT_2_ 4 wt% and (**f**) epoxy/GNP_8_SWCNT_2_ 5 wt% nanocomposite samples (top surface) after cure.

The morphology of the nanocomposites after a cure was investigated by SEM and the effect of the viscosity on the final quality of the surfaces was studied. Figure 6 shows the surface of the epoxy/GNP AO-4, epoxy/SWCNT and epoxy/GNP_8_SWCNT_2_ samples with different nanofiller loadings.

By comparing the 1 wt% systems, we note that the epoxy/GNP AO-4 sample has the smoothest surface among the analyzed specimens, followed by the epoxy/GNP_8_SWCNT_2_ and the epoxy/SWCNT sample, which exhibits the roughest surface. For the epoxy/SWCNT system, a large number of asperities are present on the specimen surface due to the formation of entanglements, which is more evident at high concentrations of nanofiller (Figure 6c). When comparing the epoxy/SWCNT with the epoxy/GNP_8_SWCNT_2_ hybrid system, we observe that the epoxy/GNP_8_SWCNT_2_ hybrid at 5 wt% (Figure 6f) shows a similar surface finish to that of epoxy/SWCNT 1 wt% system (Figure 6b). This behavior can be explained by the lower viscosity values achieved by the epoxy/GNP_8_SWCNT_2_ hybrid system, which leads to better processability and more homogenous samples. In addition, the epoxy/GNP_8_SWCNT_2_ hybrid at 5 wt% has higher electrical conductivity with respect to the epoxy/SWCNT 1 wt% (Figure 3), despite having the same SWCNT content (4 wt% of GNP AO-4 and 1 wt% of SWCNT, in a ratio of 8:2). It is evident that the graphene nanoparticles act as lubricants in the melt system, decreasing significantly the viscosity of the nanocomposite mixture. The reason behind the lower processability of the SWCNT suspensions lies in the large number of aggregates that form due to the weak interaction of the pristine SWCNTs with the epoxy matrix and to the van der Waals forces acting among the carbon nanoparticles. By contrast, in the hybrid system, graphene nanoplatelets prevent the aggregation of the single-walled nanotubes by physically hindering the process due to their large surface area. At the same time, the SWCNTs prevent the restacking of graphene nanoplatelets, resulting in more homogeneous fillers dispersion and the formation of a branched 3D network, which is essential for the enhancement of the electrical properties of the cured nanocomposites. 

**Figure 7:**
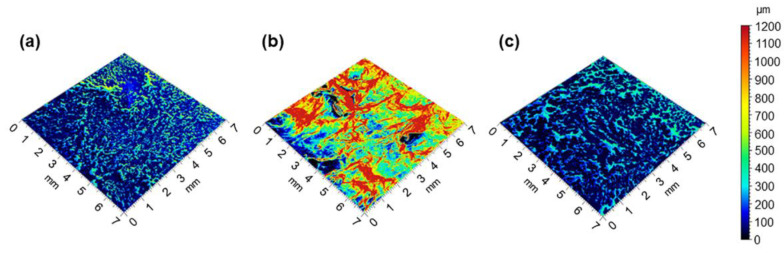
3D surface reconstruction through SEM image processing of (**a**) epoxy/SWCNT 1 wt%, (**b**) epoxy/SWCNT 4 wt% and (**c**) epoxy/GNP_8_SWCNT_2_ 4 wt% nanocomposite samples (top surface) after cure.

In order to obtain a quantitative assessment of the nanocomposite surface morphology, a 3D reconstruction with the evaluation of the surface roughness was performed using the MountainsMap software for the epoxy/SWCNT at 1 wt% and 4 wt% and for the epoxy/GNP_8_SWCNT_2_ at 4 wt% (Figure 7). The roughness analysis confirmed the visual inspection, with the epoxy/GNP_8_SWCNT_2_ at 4 wt% showing a better surface finish than the epoxy/SWCNT at 1 wt%, and with the epoxy/SWCNT at 4 wt% showing the roughest surface. The calculated mean values of surface roughness were 109.8 ± 17.5 µm and 252.1 ± 35.6 µm for the epoxy/SWCNT at 1 wt% and 4 wt%, respectively, and 97.9 ± 16.8 µm for the epoxy/GNP_8_SWCNT_2_ at 4 wt%.

## 4. Conclusions

The dispersion state and the electrical properties of RTM6 epoxy resin loaded with different grades of GNPs and with GNP/CNT hybrid nanofiller were investigated. The electrical conductivity of RTM6/GNP 1 wt% nanocomposites were shown to be of the same order of magnitude within experimental error, with the epoxy/xGNP-H5 showing the lowest electrical properties, whereas the best fillers distribution in RTM6 was achieved using the GNP AO-4 type. Next, GNP AO-4 and SWCNTs were combined in the ratio of 2:8, 5:5 and 8:2 to take advantage of the high aspect ratio of the selected carbon nanotubes and of the dispersion capabilities of GNP AO-4, creating an interconnected hybrid architecture. The electrical and rheological behavior of epoxy/SWCNT, epoxy/GNP_2_SWCNT_8_, epoxy/GNP_5_SWCNT_5_ and epoxy/GNP_8_SWCNT_2_ at different filler loadings was analyzed. Results showed that the epoxy/SWCNT system has higher electrical conductivities at all nanotube concentrations with respect to the hybrid systems, except for the epoxy/GNP_2_SWCNT_8_ at 2 wt%. However, rheological tests highlighted the large difference in viscosity for the systems analyzed, with the epoxy/hybrid mixtures having lower viscosity values at all loading fractions than the epoxy/SWCNT. In particular, the epoxy/GNP_8_SWCNT_2_ 5 wt% showed lower viscosity than the epoxy/SWCNT 1 wt%, despite having the same amount of carbon nanotubes in addition to 4 wt% of graphene nanoplatelets. This results in a net difference in the final surface quality of the samples, as revealed by SEM investigations with 3D surface reconstruction. Likewise, the epoxy/GNP_5_SWCNT_5_ 2 wt% showed similar viscosity to the epoxy/SWCNT 1 wt% despite having the same amount of carbon nanotubes in addition to 1 wt% of graphene nanoplatelets.

Regarding the electrical properties, it was observed that high loadings of the hybrid GNP_8_SWCNT_2_ mixture simultaneously ensure high electrical conductivities and low viscosity values. At 1 wt% and 2 wt% of nanofiller, the epoxy/GNP_5_SWCNT_5_ and epoxy/SWCNT nanocomposites exhibited similar electrical properties, with the epoxy/GNP_5_SWCNT_5_ hybrids maintaining viscosity values one order of magnitude lower than the SWCNT-loaded epoxy nanocomposites. The better processability along with the good electrical properties of the hybrid mixtures is attributed to the synergistic effect among the selected carbon nanoparticles: the high aspect ratio of SWCNTs is responsible for the increased electrical conductivity and for preventing the restacking of the GNPs, while the GNPs inhibit the aggregation of the SWCNTs. 

These results lead to the rational selection of the GNP/CNT ratio for the optimal processability of high-performance fiber-reinforced composites exploiting the advantages of carbon-modified matrices. In future work, liquid composite molding may be used for the production of laminates in order to analyze the enhancement of the mechanical and electrical properties of the final component. The interactions among nanoparticles and long fibers during the impregnation phase of the preform will be the subject of future investigation.

## Figures and Tables

**Table 1:** Rheology parameters of RTM6 epoxy resin with different loadings of SWCNTs and GNPs determined from non-linear least-squares fit of viscosity data (measured at 80 °C) using the power law model.

	epoxy/SWCNT				
	1 wt%	2 wt%	3 wt%	4 wt%	
*K* (Pa s^n^)	35.58	215.67	497.58	1388.81	
*n*	0.50	0.12	0.15	0.15	
$R^{2}$	0.983	0.997	0.996	0.996	
	**epoxy/GNP_2_SWCNT_8_**				
	**1 wt%**	**2 wt%**	**3 wt%**	**4 wt%**	
*K* (Pa s^n^)	20.91	176.95	360.31	851.04	
*n*	0.42	0.19	0.15	0.15	
$R^{2}$	0.991	0.999	0.999	0.999	
	**epoxy/GNP_5_SWCNT_5_**				
	**1 wt%**	**2 wt%**	**3 wt%**	**4 wt%**	
*K* (Pa s^n^)	13.40	24.28	160.51	395.54	
*n*	0.60	0.46	0.25	0.20	
$R^{2}$	0.989	0.98	0.999	0.999	
	**epoxy/GNP_8_SWCNT_2_**				
	**1 wt%**	**2 wt%**	**3 wt%**	**4 wt%**	**5 wt%**
*K* (Pa s^n^)	1.49	3.51	8.69	20.06	27.18
*n*	0.76	0.59	0.50	0.50	0.55
$R^{2}$	0.968	0.976	0.979	0.998	0.997

## Data Availability

Not applicable.
